# Some Properties of the Plaquette Random-Cluster Model

**DOI:** 10.1007/s11040-025-09543-x

**Published:** 2025-12-10

**Authors:** Paul Duncan, Benjamin Schweinhart

**Affiliations:** 1 Department of Mathematics, Indiana University, Bloomington, 47408 IN USA; 2 Department of Mathematical Sciences, George Mason University, Fairfax, 22030 VA USA

**Keywords:** Percolation, Probability, Statistical Physics, Algebraic Topology, 60K35, 82B20, 60D05, 57Z05

## Abstract

We show that the *i*-dimensional plaquette random-cluster model with coefficients in $\mathbb {Z}_q$ is dual to a $(d-i)$-dimensional plaquette random cluster model. In addition, we explore boundary conditions, infinite volume limits, and uniqueness for these models. For previously known results, we provide new proofs that rely more on the tools of algebraic topology.

## Introduction

The plaquette random-cluster model (PRCM) is a generalization of the classical random-cluster model to higher dimensions. Instead of a graph, it is a cubical complex, which we recall is a topological space built from cubes of different dimensions, sometimes also referred to as plaquettes, where two intersecting cubes are required to meet in a lower-dimensional cube. Consider a cubical complex *X* and let $X^{\left( i \right) }$ be the set of cubes of dimension *i* in *X*. We refer to $\bigcup _{k \le i} X^{\left( i \right) }$ as the *i*-skeleton of *X*. The PRCM with coefficients in $\mathbb {Z}_q$ for $q \in \mathbb {N}$ is the random *i*-dimensional subcomplex *P* of *X* containing the $(i-1)$-skeleton of *X* and a random subset of the plaquettes of $X^{\left( i \right) }$ so that

$$\begin{aligned} \mu _{X,p,q,i}\left( P \right) \propto p^{\left| P\right| }\left( 1-p \right) ^{\left| X^{\left( i \right) }\right| - \left| P\right| }\left| H^{i-1}\left( P;\;\mathbb {Z}_q \right) \right| \end{aligned}$$

where $\left| P\right|$ and $\left| X^{\left( i \right) }\right|$ denote the number of *i*-plaquettes of *P* and *X*,  respectively, and $H^{i-1}\left( P;\;\mathbb {Z}_q \right)$ is the cohomology of *P* with coefficients in $\mathbb {Z}_q.$ $\left| H^{i-1}\left( P;\;\mathbb {Z}_q \right) \right|$ counts — up to a constant factor that does not depend on *P* — the number of ways to assign spins in $\mathbb {Z}_q$ to all $(i-1)$-plaquettes of *X* that are compatible with *P* in the sense that the spins on the appropriately oriented $(i-1)$-faces incident to any single plaquette of *P* add up to zero modulo *q*. We provide a precise definition of cohomology in Sect. 2 below, and a more detailed exposition of the model and the topological notions involved can be found in Sects. 1 and 2 of [1]. This definition was first suggested in [1] (rather, an equivalent one defined in terms of homology; see Corollary 3 below), and the details were worked out independently by [2] and [3]. The PRCM is motivated by its coupling with $(i-1)$-dimensional *q*-state Potts lattice gauge theory which assigns spins to the $(i-1)$ cells of *X*; the Wilson loop expectation for an $(i-1)$-boundary $\gamma$ equals the probability that $\gamma$ is null-homologous when homology coefficients are taken in $\mathbb {Z}_q.$ When *q* is prime this PRCM coincides with the plaquette random-cluster model with coefficients in the field $\mathbb {F}_q.$ The latter was first introduced by [4], which focused on a mean-field version of the model. All results presented here also hold for the PRCM with coefficients in a field, with minor modifications.

Graphical representations have proven to be a useful tool in the study of lattice spin models such as the Potts model. For the PRCM — a cellular representation of Potts lattice gauge theory — to play the same role, its basic properties must be elucidated. The methods of [2] relied on a technical shortcut to obtain basic results about the codimension one PRCM on the way to prove a sharp phase transition for Wilson loop expectations in $(d-2)$-dimensional Potts lattice gauge theory on $\mathbb {Z}^d.$ Specifically, $(d-1)$-dimensional PRCM on $\mathbb {Z}^d$ with coefficients in an abelian group is equivalent to a PRCM with coefficients in a field, which is in turn dual to the classical 1-dimensional random-cluster model (RCM). Thus, results for the RCM concerning boundary conditions, positivity, and infinite volume limits can be translated to corresponding statements for the codimension one PRCM. We cannot rely on this logic more generally. The purpose of this paper is to prove corresponding results for general values of *i*,  including the special case of the self-dual 2-dimensional PRCM on $\mathbb {Z}^4$ which is coupled with 1-dimensional Potts lattice gauge theory. We hope that this will be helpful for researchers tackling this particularly interesting case.

One of our main goals is to show that an *i*-dimensional PRCM on $\mathbb {Z}^d$ with coefficients in $\mathbb {Z}_q$ and parameter *p* is dual to a $(d-i)$-dimensional PRCM on $\mathbb {Z}^d$ with coefficients in $\mathbb {Z}_q$ and parameter $p^*\left( p,q \right) ,$ where

$$\begin{aligned} p^*(p,q)= \frac{\left( 1-p \right) q}{\left( 1-p \right) q + p}\,. \end{aligned}$$

Note that when $i = d-i,$ this allows for the identification of a self-dual temperature. Towards that end, we study boundary conditions and infinite volume measures for the PRCM and prove a number of results about them. Some of these latter results were also shown by [3], but our proofs are shorter and employ different, more geometric arguments.

Before proceeding, we give a definition of the PRCM on a box with boundary conditions. We will explain how this generalizes the standard construction for the RCM and provide more intuition in Sect. 3. Fix *i*,  let *r* be a rectangular box in $\mathbb {Z}^d$ and let $\xi$ be a collection of *i*-plaquettes. Denote by $P_{\xi }$ the union of *P*,  the plaquettes of $\xi$ contained in $\left( \mathbb {Z}^d \setminus r \right) ,$ and the $(i-1)$-skeleton of $\mathbb {Z}^d,$ and write $\phi$ for the inclusion map from *P* into $P_{\xi }.$ Then the PRCM on *r* with boundary conditions $\xi$ is the measure $\mu _{r,p,q,i}^{\xi }$ is defined by

$$\begin{aligned} \mu _{r,p,q,i}^{\xi }\left( P \right) \propto p^{\left| P\right| }\left( 1-p \right) ^{\left| X^{\left( i \right) }\right| - \left| P\right| }\left| {{\,\textrm{im}\,}}\phi ^*\right| \,, \end{aligned}$$

where $\phi ^*$ is the map on homology induced by $\phi .$ While this does not include the important case of periodic boundary conditions, the corresponding results about that case follow by very similar arguments, some of which have already been written out in the case of *q* prime in [1] (namely, duality and positive association).

We give an informal description of our results. First, we show that if the PRCMs on a sequence of nested boxes $r_1\subset r_2\subset r_3\ldots$ constructed with boundary conditions $\xi$ converges to an infinite volume measure, then the correctly chosen dual PRCMs converge to a dual infinite volume measure. There is some subtlety in this; the dual PRCM is “wired at infinity” in a sense made precise using Borel–Moore homology. The duality theorem is proven in two steps: by establishing a finite volume analogue in Theorem 15 and then extending it to infinite volume measures in Theorem 20. We also establish a number of technical results about the PRCM with boundary conditions. Proposition 9 states that for sufficiently large boxes $\hat{r}\supset r,$ this measure coincides with the free PRCM $\hat{P}$ on $\hat{r}$ conditioned to agree with $\xi$ on $\hat{r} \setminus r.$

Next, we extend a theorem of Grimmett on the classical RCM [5] to show that there is a unique infinite volume PRCM for generic values of *p* and fixed values of *i*, *d*,  and *q* (Theorem 23). Finally, we show that finite volume (Theorem 24) and infinite volume PRCMs (Corollary 25) are positively associated using the Mayer–Vietoris sequence for homology.

## Background and Definitions

In this paper we consider random subcomplexes of the cubical complex formed by tessellating Euclidean space with translates of the unit cube $\left[ 0,1 \right] ^d.$ The *k*-dimensional cells of this complex are exactly the translates and rotations (since *k* may be less than *d* and greater than 0) of the unit cube $\left[ 0,1 \right] ^k\times \left\{ 0\right\} ^{d-k}$ which have integer corner points. We denote this complex by $\mathbb {Z}^d.$ Note that the one-skeleton of $\mathbb {Z}^d$ — the cubical complex containing the vertices and edges — is the familiar nearest neighbors graph on the integer lattice.

For a subcomplex *X*,  we write $X^{\left( k \right) }$ for the union of the cells of *X* of dimension at most *k*. If the highest dimensional cell of *X* is *k*,  then write $\left| X\right|$ for the number of *k*-cells of *X*. We say that *X* is an *i*-dimensional **percolation subcomplex** of $Y \subset \mathbb {Z}^d$ if $Y^{\left( i-1 \right) } \subset X \subset Y^{\left( i \right) }.$ In particular, any subset of the *i*-dimensional cells is permitted. We most often consider rectangular subsets of $\mathbb {Z}^d$ of the form $r = \prod _{k=1}^d\left[ a_k,b_k \right] ,$ which we call **boxes**. We will also write *r* for the union of its cells of dimension at most $(i-1)$ and the *i*-cells which intersect its interior (alternatively, we exclude the *i*-cells contained in its boundary). For the union of all cells of dimension at most *i* in *r*,  we instead write $\overline{r}.$ Sometimes it will also be convenient to work with the cube of side length 2*n*,  which we write as $\Lambda _n :=\left[ -n,n \right] ^d.$

We now provide some of the basic topological definitions used in this paper for reference, but we encourage a reader unfamiliar with topology to refer to the first appendix of [2] for a more detailed introduction. Throughout the paper, we will use the convention $\mathbb {N}= \left\{ 0,1,2,\ldots \right\}$ for the natural numbers. For $i \in \mathbb {N},$ the *i*-th **chain group** of *X* with coefficients in $\mathbb {Z}_q,$ written $C_i\left( X;\;\mathbb {Z}_q \right) ,$ is the group of $\mathbb {Z}_q$-linear combinations of oriented *i*-plaquettes of *X*. For each *i* there is a **boundary map** $\partial _i: C_i\left( X;\;\mathbb {Z}_q \right) \rightarrow C_{i-1}\left( X;\;\mathbb {Z}_q \right)$ which is linear and maps a plaquette to an oriented sum of its $(i-1)$-faces so that $\partial _{i-1} \circ \partial _i = 0$ for each $i \in \mathbb {N}.$ When the dimension is understood, the subscript is often dropped and the boundary map is just written as $\partial .$ The *i*-th **cycle group** is defined by $Z_i\left( X;\;\mathbb {Z}_q \right) :=\ker \partial _{i} \subset C_i\left( X;\;\mathbb {Z}_q \right)$ and the *i*-th **boundary group** is defined by $B_i :={{\,\textrm{im}\,}}\partial _{i+1} \subset C_i\left( X;\;\mathbb {Z}_q \right) .$ Then the *i*-th **homology group** is defined by $H_i\left( X;\;\mathbb {Z}_q \right) :=Z_i\left( X;\;\mathbb {Z}_q \right) / B_i\left( X;\;\mathbb {Z}_q \right) .$

As an example, let $\sigma _1,\ldots ,\sigma _6$ be the two-dimensional faces of the unit cube $\left[ 0,1 \right] ^3$ in $\mathbb {Z}^3$ and let *X* be the two-complex obtained by removing $\sigma _5$ and $\sigma _6.$ Then $Z_1\left( X;\;\mathbb {Z}_q \right)$ is generated by $\partial \sigma _1,\ldots ,\partial \sigma _5$ (note that $\partial \sigma _6=\partial \sigma _1+\ldots +\partial \sigma _5$ when the faces are oriented appropriately) and $Z_1\left( X;\;\mathbb {Z}_q \right) \cong \left( \mathbb {Z}_q \right) ^5.$ Also, $B_1\left( X;\;\mathbb {Z}_q \right) \cong \left( \mathbb {Z}_q \right) ^{4}$ as it is generated by the boundaries of the four 2-cells of *X*. Thus $H_1\left( X;\;\mathbb {Z}_q \right) \cong \mathbb {Z}_q$ has rank one, which matches our intuition that the first homology should count the number of “one-dimensional holes” of *X*. If we add $\sigma _5$ to *X*,  it kills the remaining generator of $H_1\left( X;\;\mathbb {Z}_q \right) .$ The addition of $\sigma _6$ has the effect of adding a generator to the two-dimensional homology. When *q* is prime, it is true in general that the addition of a single *i*-plaquette either increases the rank of the *i*-dimensional homology by one or decreases the rank of the $(i-1)$-dimensional homology by one. However, this is not true in general when *q* is prime, nor is it always the case that the rank of the homology only depends on *q* (though there are no examples embeddable in $\mathbb {Z}^3$). For example, there is subcomplex $\mathbb {R}P^2$ of $\mathbb {Z}^4$ homeomorphic to the real projective plane, a space by attaching a disk to the boundary of the Möbius strip. $\mathbb {R}P^2$ is an example of a non-orientable surface, which means that the 2-cells of $\mathbb {R}P^2$ cannot be oriented so that every edge appears in the boundary of one cell with positive orientation and one cell with negative orientation. This implies that $Z_2\left( \mathbb {R}P^2;\;\mathbb {Z}_q \right) \cong H_2\left( \mathbb {R}P^2;\;\mathbb {Z}_q \right) \cong 0$ when *q* is odd. However, every edge is incident to exactly two faces so if $\sigma _1,\ldots ,\sigma _k$ then $\partial \left( \sigma _1+\ldots +\sigma _k \right) =0\in C_1\left( \mathbb {R}P^2;\;\mathbb {Z}_2 \right)$ so $\sigma _1+\ldots +\sigma _k\in Z_2\left( \mathbb {R}P^2;\;\mathbb {Z}_2 \right) .$ It’s easy to see that this is the only non-zero element of $Z_2\left( \mathbb {R}P^2;\;\mathbb {Z}_2 \right)$ so $Z_2\left( \mathbb {R}P^2;\;\mathbb {Z}_2 \right) \cong H_2\left( \mathbb {R}P^2;\;\mathbb {Z}_2 \right) \cong \mathbb {Z}_2.$ More generally, we have the following.

$$\begin{aligned} H_0\left( \mathbb {R}P^2;\;\mathbb {Z}_q \right)&\cong \mathbb {Z}_q\\ H_1\left( \mathbb {R}P^2;\;\mathbb {Z}_q \right)&\cong {\left\{ \begin{array}{ll} 0 &  q\in 2\mathbb {N}+3\\ \mathbb {Z}_2 &  q\in 2\mathbb {N}+2\end{array}\right. }\\ H_2\left( \mathbb {R}P^2;\;\mathbb {Z}_q \right)&\cong {\left\{ \begin{array}{ll} 0 &  q\in 2\mathbb {N}+3\\ \mathbb {Z}_2 &  q\in 2\mathbb {N}+2\end{array}\right. }\,.\\ \end{aligned}$$

Also, observe that the addition of $\sigma _k$ to $\mathbb {R}P^2 \setminus \sigma _k$ increases the size of the 2-homology when *q* is even but reduces the size of the 1-homology when *q* is odd.

We also use the algebraic dual of these notions, which are indicated with superscripts instead of subscripts. The *i*-th cochain group is the group of homomorphisms from chains to $\mathbb {Z}_q,$ written as $C^i\left( X;\;\mathbb {Z}_q \right) :=\textrm{Hom}\left( H_i\left( X;\;\mathbb {Z}_q \right) ,\mathbb {Z}_q \right) .$ The cochain groups come with **coboundary maps** $\delta ^i: C^i\left( X;\;\mathbb {Z}_q \right) \rightarrow C^{i+1}\left( X;\;\mathbb {Z}_q \right)$ which satisfy $\left( \delta _i \alpha \right) \left( \bullet \right) = \alpha \left( \delta _{i+1} \bullet \right)$ for any $\alpha \in C^i\left( X;\;\mathbb {Z}_q \right) .$ It follows immediately that $\delta ^i \circ \delta ^{i-1} = 0$ for each $i \in \mathbb {N}.$ Then there are the analogous **cocycle**, **coboundary**, and **cohomology groups** defined by $Z^i\left( X;\;\mathbb {Z}_q \right) :=\ker \delta ^i,$ $B^i\left( X;\;\mathbb {Z}_q \right) :={{\,\textrm{im}\,}}\delta ^{i-1},$ and $H^i\left( X;\;\mathbb {Z}_q \right) :=Z^i\left( X;\;\mathbb {Z}_q \right) / B^i\left( X;\;\mathbb {Z}_q \right)$ respectively. In the context of Potts lattice gauge theory, $B^i\left( X;\;\mathbb {Z}_q \right)$ is called the *gauge group*. As we will see below, if $g\in B^i\left( X;\;\mathbb {Z}_q \right)$ then the operation $f\mapsto f+g$ leaves the Hamiltonian of PLGT unchanged. Moreover, if *P* is an *i*-dimensional percolation subcomplex of *X* then the elements of $Z^i\left( P;\;\mathbb {Z}_q \right)$ are precisely the cochains compatible with *P* in the sense defined above and $B^i\left( P;\;\mathbb {Z}_q \right) =B^i\left( X;\;\mathbb {Z}_q \right)$ (since *P* contains all $(i-1)$-faces) does not depend on *P*. Thus

$$\begin{aligned} \left| H^i\left( P;\;\mathbb {Z}_q \right) =\frac{\left| Z^i\left( P;\;\mathbb {Z}_q \right) \right| }{\left| B^i\left( X;\;\mathbb {Z}_q \right) \right| }\right| \end{aligned}$$

counts the number of spin assignments compatible with *P* up to a factor which depends only on *X*. Returning to the example from the previous paragraph, we have that

$$\begin{aligned} H^0\left( \mathbb {R}P^2;\;\mathbb {Z}_q \right)&\cong \mathbb {Z}_q\\ H^1\left( \mathbb {R}P^2;\;\mathbb {Z}_q \right)&\cong {\left\{ \begin{array}{ll} 0 &  q\in 2\mathbb {N}+3\\ \mathbb {Z}_2 &  q\in 2\mathbb {N}+2\end{array}\right. }\\ H^2\left( \mathbb {R}P^2;\;\mathbb {Z}_q \right)&\cong {\left\{ \begin{array}{ll} 0 &  q\in 2\mathbb {N}+3\\ \mathbb {Z}_2 &  q\in 2\mathbb {N}+2\end{array}\right. }\,.\\ \end{aligned}$$

It will also be useful later on to recall that an inclusion map $\phi : X \hookrightarrow Y$ can be used to include plaquettes from *X* into *Y* and thus gives a map on the chain groups $C_i\left( X;\;\mathbb {Z} \right) \rightarrow C_i\left( Y;\;\mathbb {Z}. \right)$ It is then straightforward to check that this induces a map on homology $\phi _*: H_i\left( X;\;\mathbb {Z} \right) \rightarrow H_i\left( Y;\;\mathbb {Z} \right) .$ By the same token, cochains on *Y* can be restricted to *X*,  which induces a map $\phi ^*: H^i\left( Y;\;\mathbb {Z} \right) \rightarrow H^i\left( X;\;\mathbb {Z} \right) .$

### The PRCM and PLGT

The *i*-dimensional plaquette random-cluster model (PRCM) of a finite subcomplex $X \subset \mathbb {Z}^d$ with parameters $p \in \left[ 0,1 \right] ,$ $q \in \mathbb {N}+2$ is defined as follows:

#### Definition 1

Let $\mu _{X,p,q,i}$ be the measure on percolation subcomplexes $P \subset X$ given by

$$\begin{aligned} \mu _{X,p,q,i} \propto p^{\left| P\right| }\left( 1-p \right) ^{\left| X^{\left( i \right) }\right| - \left| P\right| }\left| H^{i-1}\left( P;\;\mathbb {Z}_q \right) \right| \,. \end{aligned}$$

The PRCM can equivalently be defined in terms of homology rather than cohomology as a consequence of the universal coefficient theorem. We recall a formulation given in [2].

#### Proposition 2

If $H_{j-2}\left( P;\;\mathbb {Z}_q \right)$ vanishes (or, more generally, is a free $\mathbb {Z}_q$-module) then the map $h:H^{j-1}\left( P;\;\mathbb {Z}_q \right) \rightarrow \textrm{Hom}\left( H_{j-1}\left( P;\;\mathbb {Z}_q \right) ,\mathbb {Z}_q \right)$ given by evaluating a cocycle on a homology class is an isomorphism. In particular,

$$\begin{aligned} \left| H^{j-1}\left( P;\;\mathbb {Z}_q \right) \right| =\left| H_{j-1}\left( P;\;\mathbb {Z}_q \right) \right| \,. \end{aligned}$$

This implies that $H^{i-1}\left( P;\;\mathbb {Z}_q \right) \cong H_{i-1}\left( P;\;\mathbb {Z}_q \right)$ for an *i*-dimensional percolation subcomplex *P* of a box. In fact, the universal coefficient theorems for homology and cohomology imply a stronger result, which we state without proof here since we do not need it below: if $\textbf{b}_j\left( P \right) ={{\,\textrm{rank}\,}}H_j\left( P;\;\mathbb {Z} \right)$ denotes the Betti number then there is a finite group *T* so that

$$\begin{aligned} H^{i}\left( P;\;\mathbb {Z}_q \right) \cong H_{i}\left( P;\;\mathbb {Z}_q \right) \cong \left( \mathbb {Z}_q \right) ^{\textbf{b}_{i}\left( P \right) }\oplus T \end{aligned}$$

and

$$\begin{aligned} H^{i-1}\left( P;\;\mathbb {Z}_q \right) \cong H_{i-1}\left( P;\;\mathbb {Z}_q \right) \cong \left( \mathbb {Z}_q \right) ^{\textbf{b}_{i-1}\left( P \right) }\oplus T\,. \end{aligned}$$

#### Corollary 3

Define

$$\begin{aligned} \hat{\mu }_{X,p,q,i} \propto p^{\left| P\right| }\left( 1-p \right) ^{\left| X^{\left( i \right) }\right| - \left| P\right| }\left| H_{i-1}\left( P;\;\mathbb {Z}_q \right) \right| \,. \end{aligned}$$

Then for any box $r \subset \mathbb {Z}^d,$

$$\begin{aligned} \mu _{r,p,q,i} \,{\buildrel d \over =}\, \hat{\mu }_{r,p,q,i}\,. \end{aligned}$$

As a result, we may either work with the homology or cohomology of *P* as is convenient. The definition involving cohomology is more natural in the context of the coupling of the PRCM with Potts lattice gauge theory (PLGT), which we now describe.

Recall that the $(i-1)$-dimensional *q*-state PLGT with inverse temperature parameter $\beta$ on a finite subset $X \subset \mathbb {Z}^d$ is the random element of $C^{i-1}\left( X;\;\mathbb {Z}_q \right)$ distributed according to

$$\begin{aligned} \nu _{X,\beta ,q,k}\left( f \right) \propto e^{-\beta H\left( f \right) }\,, \end{aligned}$$

where *H* is the Hamiltonian defined by

1 $$\begin{aligned} H\left( f \right) =-\sum _{\sigma }K\left( \delta f\left( \sigma \right) ,0 \right) \,. \end{aligned}$$

Here *K* is the Kronecker delta function and $\delta$ is the coboundary operator.

The PRCM and PLGT can be coupled in a fashion analogous to the Edwards–Sokal coupling of the classical random-cluster model and the Potts model [6, 7]. This was proven for general *q* independently in [2] and [3].

#### Theorem 4

( [2, 3]) Let *X* be a finite cubical complex, $q\in \mathbb {N}+1,$ $\beta \in [0,\infty ),$ and $p = 1-e^{-\beta }.$ Define a coupling on $C^{i-1}\left( X \right) \times \left\{ 0,1\right\} ^{X^{\left( i \right) }}$ by

$$\begin{aligned} \kappa \left( f,P \right) \propto \prod _{\sigma \in X^{\left( i \right) }}\left[ \left( 1-p \right) I_{\left\{ \sigma \notin P\right\} } + p I_{\left\{ \sigma \in P,\delta f\left( \sigma \right) =0\right\} } \right] \,. \end{aligned}$$

Then $\kappa$ has the following marginals.

- The first marginal is $\nu _{X,\beta ,q,i-1}.$
- The second marginal is $\mu _{X,p,q,i}.$

The most important observable in PLGT is the Wilson loop variable, which can be calculated by summing the spins on the edges of a loop and outputting the corresponding *q*-th root of unity. Since the loops can be thought of as cycles, it is therefore most natural in the context of Wilson loop variables to view spin assignments as functions on those cycles, i.e. cocycles. In fact, from this viewpoint the gauge transformations are just the coboundaries, leading to a natural description of PLGT in terms of cohomology. Then using the coupling given in Theorem 4, the term $\left| H^{i-1}\left( P;\;\mathbb {Z}_q \right) \right|$ in the definition of the PRCM can be viewed as counting equivalence classes of spin assignments to the $(i-1)$-faces of *X*.

## Boundary Conditions

First, consider the familiar random-cluster model on a graph. A boundary condition on a subgraph $S \subset \mathbb {Z}^d$ induced by some finite vertex set can be thought of as a configuration of edges not contained in *S*.

Let $\xi$ be a set of edges in $\mathbb {Z}^d$ and write $P^{\xi }$ for the set of open edges of $\xi \cap \left( \mathbb {Z}^d \setminus S \right) .$ The idea is to define a random-cluster measure on *S* with the additional edges of $P^{\xi }$ added for the purpose of counting connected components. Of course, $P^{\xi }$ will have infinitely many connected components in general, but finitely many of them are connected to *S*.

More precisely, there is a corresponding random-cluster measure on *S* with boundary condition $\xi$ written as $\mu _{S,p,q,1}^{\xi }\left( P \right) ,$ where the term $\textbf{b}_0\left( P \right)$ counting the number of connected components of *P* in *S* is replaced by the number of connected components of $P_{\xi }$ that intersect *S*. The extremal cases of $\xi$ containing all closed or all open edges are called free and wired boundary conditions respectively, and we write $\mu _{S,p,q,1}^{\textbf{f}}$ and $\mu _{S,p,q,1}^{\textbf{w}}$ for the associated measures.

Boundary conditions in the PRCM on $\mathbb {Z}^d$ are defined analogously, in that we want to define a random-cluster model on a subcomplex *X* with the additional topological information from external plaquettes. Let $\xi$ be a set of plaquettes and recall that $P_{\xi } = P \cup \left( \xi \cap \left( \mathbb {Z}^d \setminus X \right) \right) \cup \bigcup _{k=1}^{i-1}\left( \mathbb {Z}^d \right) ^{\left( k \right) }$ is the percolation subcomplex that agrees with *P* within *X* and with $\xi$ outside of *X* (the last term is just the lower dimensional cells that are included in every percolation subcomplex).

### Definition 5

Let $\phi : P \rightarrow P_{\xi }$ be the inclusion map and let

$$\begin{aligned} \phi ^* : H^{i-1}\left( P_{\xi };\;\mathbb {Z}_q \right) \rightarrow H^{i-1}\left( P;\;\mathbb {Z}_q \right) \end{aligned}$$

be the induced map on cohomology. The measure $\mu _{X,p,q,i}^{\xi }$ is defined by

$$\begin{aligned} \mu _{X,p,q,i}^{\xi }\left( P \right) \propto p^{\left| P\right| }\left( 1-p \right) ^{\left| X^{\left( i \right) }\right| - \left| P\right| }\left| {{\,\textrm{im}\,}}\phi ^*\right| \,. \end{aligned}$$

Note that taking $i=1$ recovers the definition for the classical RCM because in that case $\left| {{\,\textrm{im}\,}}\phi ^*\right|$ counts the number of connected components of $P_{\xi }$ that intersect *P*. To get a feel for what this definition means, we consider the examples of free and wired boundary conditions. In the former case, $\phi ^*$ is surjective because there are no additional boundaries and

$$\begin{aligned} {{\,\textrm{im}\,}}\phi ^* = H^{i-1}\left( P;\;\mathbb {Z}_q \right) \end{aligned}$$

so the measure (denoted $\mu _{X,p,q,i}^{\textbf{f}}$) coincides with the earlier definition of the random-cluster measure on the finite complex *X*. On the other hand, as long as $H^{i-1}\left( \mathbb {Z}^d \setminus X;\;\mathbb {Z}_q \right) = 0,$ an element of $H^{i-1}\left( P_{\xi };\;\mathbb {Z}_q \right)$ must vanish on $(i-1)$-cycles supported on the boundary of *X* when we use wired boundary conditions. The wired measure $\mu _{X,p,q,i}^{\textbf{w}}$ is then the same as the finite volume random-cluster measure on *X* with the boundary $(i-1)$-cells all identified. Specifically, when $X=r$ is a box in $\mathbb {Z}^d,$ we have that

$$\begin{aligned} {{\,\textrm{im}\,}}\phi ^* \cong H^{i-1}\left( P \cup \partial r;\;\mathbb {Z}_q \right) \,, \end{aligned}$$

where we abuse notation and write $\partial r$ for the topological boundary of *r*,  i.e. the union of its facets. The term on the right will appear again when we discuss Alexander duality.

An analogue of Corollary 3 on the equivalence of homological and cohomological perspectives also holds for the PRCM with boundary conditions.

### Lemma 6

Let $P_1 \subset P_2$ be percolation complexes, and let $\phi ^*:H^{i-1}\left( P_2;\;\mathbb {Z}_q \right) \rightarrow H^{i-1}\left( P_1;\;\mathbb {Z}_q \right)$ and $\phi _*:H^{i-1}\left( P_1;\;\mathbb {Z}_q \right) \rightarrow H^{i-1}\left( P_2;\;\mathbb {Z}_q \right)$ be the homomorphisms induced by the inclusion $\phi :P_1\hookrightarrow P_2.$ Then

$$\begin{aligned} \left| {{\,\textrm{im}\,}}\phi ^*\right| = \left| {{\,\textrm{im}\,}}\phi _*\right| \,. \end{aligned}$$

In particular,

$$\begin{aligned} \mu _{X,p,q,i}^{\xi }\left( P \right) \propto p^{\left| P\right| }\left( 1-p \right) ^{\left| X^{\left( i \right) }\right| - \left| P\right| }\left| {{\,\textrm{im}\,}}\phi ^*\right| = p^{\left| P\right| }\left( 1-p \right) ^{\left| X^{\left( i \right) }\right| - \left| P\right| }\left| {{\,\textrm{im}\,}}\phi _*\right| \,. \end{aligned}$$

### Proof

We have the following commutative diagram. 
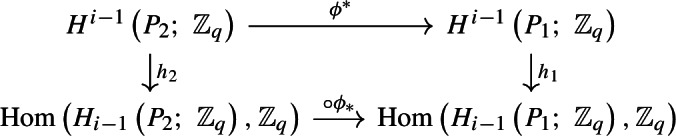


Here, $h_j:H^{i-1}\left( P_j;\; \mathbb {Z}_q \right) \rightarrow \textrm{Hom}\left( H_{i-1}\left( P_j;\;\mathbb {Z}_q \right) ,\mathbb {Z}_q \right)$ is the homomorphism induced by sending $\left[ f \right] \in H^{i-1}\left( P_j;\; \mathbb {Z}_q \right)$ to the homomorphism that sends $\left[ \sigma \right] \in H_{i-1}\left( P_j;\;\mathbb {Z}_q \right)$ to $f\left( \sigma \right)$ (this is well-defined by standard arguments; see Section 3.1. of [8]). The maps $h_j$ are in fact isomorphisms by Proposition 2. In addition, the lower horizontal row sends a homomorphism $f\in \textrm{Hom}\left( H_{i-1}\left( P_2;\;\mathbb {Z}_q \right) ,\mathbb {Z}_q \right)$ to $f\circ \phi _* \in \textrm{Hom}\left( H_{i-1}\left( P_1;\;\mathbb {Z}_q \right) ,\mathbb {Z}_q \right) .$ By following the diagram, we have that

$$\begin{aligned} {{\,\textrm{im}\,}}\phi ^* \cong \textrm{Hom}\left( \phi _*\left( H_{i-1}\left( P_1;\;\mathbb {Z}_q \right) \right) ,\mathbb {Z}_q \right) \end{aligned}$$

and, in particular,

$$\begin{aligned} \left| {{\,\textrm{im}\,}}\phi ^*\right| = \left| \textrm{Hom}\left( \phi _*\left( H_{i-1}\left( P_1;\;\mathbb {Z}_q \right) \right) ,\mathbb {Z}_q \right) \right| =\left| {{\,\textrm{im}\,}}\phi _*\right| \,. \end{aligned}$$

$$\square $$

For finitely supported boundary conditions, there is a straightforward relationship between dual boundary conditions in terms of the definitions that we have already provided. However, the general case is more subtle, and in order to state Theorem 15 in full generality, we will also want a notion of boundary conditions that are “wired at infinity.” This will require additional algebraic machinery to define so we begin by motivating it with a comparison to the situation for free boundary conditions. Consider the approximation of $\xi$ given by $\xi \cap r$ for a large box *r*. This can be thought of as an approximation of $\xi$ with free boundary conditions outside of *r*. We will soon see that $\mu _{X,p,q,i}^{\xi } =\mu _{X,p,q,i}^{\xi \cap r}$ for sufficiently large *r*. One could just as easily consider a wired approximation in which we add all plaquettes outside of *r* to $\xi .$ This also converges, but to boundary conditions which are “wired at infinity” as defined below. To see that the limits may be different, consider the classical random-cluster model in a box with boundary conditions that contain two disjoint infinite paths meeting the boundary of the box at vertices *v* and *w*. Clearly, *v* and *w* are externally connected in any wired approximation of the type described above and externally disconnected in any free approximation.

To define boundary conditions which are wired at infinity, it will be more convenient to work with homology rather than cohomology. In order to capture the limit of wired approximations, it is natural to consider cycles that “pass through infinity” in some sense. One way to formalize this is using Borel–Moore homology, for which an exposition of the viewpoint we use here can be found in Chapter 3 of [9]. Recall that Borel–Moore homology of a space *X* with *i*-cells $X^{\left( i \right) }$ can be defined in terms of the locally finite chain groups

$$\begin{aligned} C_k^{\textrm{BM}}\left( X;\;\mathbb {Z}_q \right) :=\left\{ \sum _{\sigma \in X^{\left( k \right) }} a_{\sigma } \sigma : a_{\sigma } \in \mathbb {Z}_q\right\} \,.\end{aligned}$$

The important difference between these and the usual chain groups is that the sum is permitted to have infinitely many nonzero terms. The usual boundary operator can then be extended linearly to obtain

$$\begin{aligned} \partial _k^{\textrm{BM}} : C_k^{\textrm{BM}}\left( X;\;\mathbb {Z}_q \right) \rightarrow C_{k-1}^{\textrm{BM}}\left( X;\;\mathbb {Z}_q \right) \,, \end{aligned}$$

and then the homology is given by

$$\begin{aligned} H_k^{\textrm{BM}}\left( X;\;\mathbb {Z}_q \right) :=Z_k^{\textrm{BM}}\left( X;\;\mathbb {Z}_q \right) / B_k^{\textrm{BM}}\left( X;\;\mathbb {Z}_q \right) \,, \end{aligned}$$

where $Z_k^{\textrm{BM}}\left( X;\;\mathbb {Z}_q \right) :=\ker \partial _k^{\textrm{BM}}$ and $B_k^{\textrm{BM}}\left( X;\;\mathbb {Z}_q \right) :={{\,\textrm{im}\,}}\partial _{k+1}^{\textrm{BM}},$ analogous to the classical homological definitions. For example, $H_d\left( \mathbb {Z}^d;\;\mathbb {Z}_q \right) =0$ but the oriented sum of all *d*-cells of $\mathbb {Z}^d$ is a non-trivial Borel–Moore cycle and $H_d^{\textrm{BM}}\left( \mathbb {Z}^d;\;\mathbb {Z}_q \right) \cong \mathbb {Z}_q.$ Also, $H_0\left( \mathbb {Z}^d;\;\mathbb {Z}_q \right) \cong \mathbb {Z}_q$ and $H_0^{\textrm{BM}}\left( \mathbb {Z}^d;\;\mathbb {Z}_q \right) =0.$ Finally, returning to the example with infinite paths extending from *v* and *w*,  there is a Borel–Moore chain with boundary $v-w,$ namely the (infinite) sum of the edges in the two paths.

### Definition 7

As before, denote by $\phi : P \rightarrow P_{\xi }$ the inclusion map. Also, let

$$\begin{aligned} \phi _*^{\textrm{BM}} : H_{i-1}^{\textrm{BM}}\left( P;\;\mathbb {Z}_q \right) \rightarrow H_{i-1}^{\textrm{BM}}\left( P_{\xi };\;\mathbb {Z}_q \right) \end{aligned}$$

be the induced map on Borel–Moore homology. Then define

$$\begin{aligned} \mu _{X,p,q,i}^{\overline{\xi }}\left( P \right) \propto p^{\left| P\right| }\left( 1-p \right) ^{\left| X^{\left( i \right) }\right| - \left| P\right| }\left| {{\,\textrm{im}\,}}\left( \phi _*^{\textrm{BM}} \right) \right| \,. \end{aligned}$$

This is a slight abuse of notation, since we have not defined $\overline{\xi }$ by itself, but we only write it in the context of this measure. Note that if $\xi$ contains all but finitely many plaquettes of $\mathbb {Z}^d$ then $\mu _{X,p,q,i}^{\overline{\xi }}{\buildrel d \over =} \mu _{X,p,q,i}^{\xi }.$ We will show later (in Proposition 9) boundary conditions of the form $\bar{\xi }$ can be modified so that this is the case. Unlike Definitions 1 and 5, Definition 7 does not have an immediate cohomological version.

These are not the only possible definitions of boundary conditions. It is common to consider boundary conditions of the Potts model defined by specifying boundary spins directly, which leads to a more general notion than we consider here. Various subsets of these types of conditions are defined in [3], where they are studied as subgroups of the full group of possible spin states using elementary group theory. As we are motivated by the limiting measure in $\mathbb {Z}^d,$ we will restrict ourselves to those which are compatible with some configuration of external plaquettes under the coupling, referred to as “imprint boundary conditions” in [3]. Since these arise from concrete cubical complexes, this allows us to take a more geometric approach. Notice that the special case of constant spins on the boundary of the domain does arise from an external plaquette configuration because it is equivalent to wired boundary conditions up to a choice of gauge (in topological terms, up to a choice of coboundary).

We now show that the effects of an infinite volume boundary condition appear in sufficiently large finite approximations. We first prove a straightforward characterization of nullhomology in the Borel-Moore setting in terms of finite approximations. Although we work with Borel-Moore chains to streamline the proof, we remark that the second condition in the lemma is equivalent to an analogous one for ordinary homology.

### Lemma 8

Let $X \subset \mathbb {Z}^d$ be an *i*-dimensional percolation subcomplex, and let $\xi$ be boundary conditions. Then for any finitely supported $\gamma \in Z^{\textrm{BM}}_{i-1}\left( X;\;\mathbb {Z}_q \right) ,$ $0 = \left[ \gamma \right] \in H^{\textrm{BM}}_{i-1}\left( X;\;\mathbb {Z}_q \right)$ if and only if there is an $N \in \mathbb {N}$ so that for all $n>N,$ $\gamma$ is homologous to an $(i-1)$-cycle $\gamma _n$ supported on $\partial \Lambda _n.$

### Proof

The forward implication is obvious, so suppose that for each $n>N$ there exists a $\tau _n\in C_i\left( X \cap \Lambda _n;\;\mathbb {Z}_q \right)$ so that

2 $$\begin{aligned} \partial \tau _n = \gamma +\gamma _n \end{aligned}$$

where $\gamma _n$ is supported on $\partial \Lambda _n.$ By a standard argument, we can choose the chains $\tau _n$ to be compatible in the sense that the restriction of $\tau _m$ to $\Lambda _n$ is $\tau _n$ for $m>n.$ Say that $\tau _{n_0}\in C^{\textrm{BM}}_i\left( X\cap \Lambda _{n_0};\;\mathbb {Z}_q \right)$ is *extendable* if there exist infinitely many $n_1>n_0$ and $\tau _{n_1}\in C^{\textrm{BM}}_i\left( X\cap \Lambda _{n_1};\;\mathbb {Z}_q \right)$ which satisfy (2) and so that

$$\begin{aligned} \tau _{n_1}=\tau _{n_0}+\eta _{n_1,n_0} \end{aligned}$$

for some $\eta _{n_1,n_0}$ supported outside of $\Lambda _{n_0}.$ As there are only finitely many choices of $\tau _{n_0}$ and the restriction of a chain satisfying that equation for a larger value of *n* satisfies it for $n_0,$ there exists at least one extendable choice of $\tau _{n_0}.$ Suppose *n* is large enough so that that $\gamma$ is supported on $\Lambda _{n}$ and choose an extendable chain $\tau _n.$ Since $\tau _n$ is extendable, there must exist an extendable choice of $\tau _{n+1}\in C^{\textrm{BM}}_i\left( X\cap \Lambda _{n+1};\;\mathbb {Z}_q \right)$ whose restriction to $\Lambda _n$ is $\tau _n.$ Continuing this construction one step at a time for all $m>n$ results in the desired compatible family of chains $\left\{ \tau _m\right\} _{m\ge n}.$ Setting $\eta _{n}=\tau _{m+1}-\tau _m$ we obtain that

$$\begin{aligned} \eta :=\sum _{m=n}^{\infty } \eta _n \end{aligned}$$

is an element of $C^{\textrm{BM}}_i\left( X;\;\mathbb {Z}_q \right)$ so that $\partial \eta =\gamma .$ $\square$

Given boundary conditions $\xi$ and a subcomplex $X \subset \mathbb {Z}^d,$ define

$$\begin{aligned} \xi _{X} :=\xi \cap X \end{aligned}$$

and

$$\begin{aligned} \hat{\xi }_X :=\xi \cup \left( \mathbb {Z}^d \setminus X \right) ^{\left( i \right) }\,. \end{aligned}$$

Note that for a sufficiently large box *r*,  $\hat{\xi }_X$ and $\hat{\xi }_X \cap r$ have the same effect as boundary conditions for *X*. We now use this observation to show that any boundary condition, including one of the type introduced in Definition 7, can be replaced by a finite boundary condition from Definition 5. After that, we will see that any of these measures can be obtained from one of the form given in Definition 1 by conditioning.

### Proposition 9

Let *r* be a box in $\mathbb {Z}^d$ and let $\xi$ be boundary conditions for PRCM on *r*. Then there is a cube $\Lambda _n$ containing *r* so that for any $r' \supset \Lambda _n,$

$$\begin{aligned} \mu _{r ,p,q,i}^{\xi } {\mathop {=}\limits ^{d}} \mu _{r ,p,q,i}^{\xi _{r'}}\, \end{aligned}$$

and

$$\begin{aligned} \mu _{r ,p,q,i}^{\overline{\xi }} {\mathop {=}\limits ^{d}} \mu _{r ,p,q,i}^{\hat{\xi }_{r'}}\, \end{aligned}$$

### Proof

We first prove the statement for $\mu _{r,p,q,i}^{\xi }.$ Fix a percolation subcomplex *P* of *r*. Here we will view the cluster weight term as homological rather than cohomological by applying Lemma 6 (so that we do not need to switch perspectives for the proof of the second statement). Roughly speaking, our goal is to show that $\left| {{\,\textrm{im}\,}}\phi _*\right|$ is determined by some finite subcomplex of $P_{\xi },$ where

$$\begin{aligned} \phi _*:H_{i-1}\left( P;\;\mathbb {Z}_q \right) \rightarrow H_{i-1}\left( P_{\xi };\;\mathbb {Z}_q \right) \end{aligned}$$

is the homomorphism induced by the inclusion $P\hookrightarrow P_{\xi }.$

For each $\gamma \in \ker \phi _*,$ we can find a chain $\tau _{\gamma } \in C_i\left( P_{\xi };\;\mathbb {Z}_q \right)$ so that $\partial \tau _{\gamma } = \gamma .$ By definition, $\tau _{\gamma }$ is supported on finitely many *i*-plaquettes, and therefore on $\Lambda _n \cap P_{\xi }$ for a sufficiently large *n*. In fact, we may choose *n* to be large enough so that for all $\gamma \in \ker \phi _*,$ $\tau _{\gamma }$ is supported on $\Lambda _n \cap P_{\xi }.$ Given $r' \supset \Lambda _n,$ define

$$\begin{aligned} \phi ^{r'}_*:H_{i-1}\left( P;\; \mathbb {Z}_q \right) \rightarrow H_{i-1}\left( r' \cap P_{\xi };\;\mathbb {Z}_q \right) \end{aligned}$$

to be the map on inclusion. From the first isomorphism theorem we have that

$$\begin{aligned} \ker \phi _*=\ker \phi ^{r'}_*\implies \left| {{\,\textrm{im}\,}}\phi _*\right| = \left| {{\,\textrm{im}\,}}\phi ^{r'}_*\right| \,. \end{aligned}$$

Since there are only finitely many percolation subcomplexes *P* of *r*,  this equality holds for all of them when *n* is sufficiently large. We can therefore find an *n* so that

$$\begin{aligned} \mu _{r ,p,q,i}^{\xi } {\mathop {=}\limits ^{d}} \mu _{r ,p,q,i}^{\xi _{r'}} \end{aligned}$$

for all $$r' \supset \Lambda _n.$$

The case of $\mu _{r,p,q,i}^{\overline{\xi }}$ is similar in spirit. Notice that the size of the kernel of the induced map on Borel–Moore homology

$$\begin{aligned} \phi _*^n:H^{\textrm{BM}}_{i-1}\left( P;\;\mathbb {Z}_q \right) \rightarrow H^{\textrm{BM}}_{i-1}\left( P_{\hat{\xi }_{\Lambda _n} ;\;\mathbb {Z}_q} \right) \end{aligned}$$

is decreasing in *n*. As such, it suffices to show that there is an *n* so that $\ker \phi _*^n\subseteq \ker \phi _*^{\textrm{BM}}.$ It follows from Lemma 8 that for any fixed *P*,  $\gamma \in \ker \phi _*^{\textrm{BM}}$ if and only if $\gamma \in \ker \phi _*^n$ for all sufficiently large *n*. Then, as there are only finitely many choices of *P* and $\gamma ,$ we can choose *n* large enough so that this is true for all of them. $\square$

Although it is not important in the context of this paper, we remark that although there is not an obvious analogue of Lemma 6 for $\mu ^{\overline{\xi }}_{r,p,q,i},$ one can use Proposition 9 to give an alternative definition as a limit of measures defined via cohomology.

These results allow us to give an alternative topological proof of the fact that boundary conditions are compatible with conditioning on subcomplexes, which appears as Proposition 60 of [3]. We are also able to generalize slightly to the wired boundary conditions of Definition 7.

### Corollary 10

Let $r_1\subset r_2$ be boxes in $\mathbb {Z}^d$ and let $\xi _2$ be boundary conditions for $r_2.$ For a subset $\xi _1$ of the *i*-plaquettes of $r_2\setminus r_1$ let $A\left( \xi _1 \right)$ be the event that $P\cap r_2\setminus r_1=\xi _1.$ Then, if

$$\begin{aligned} \mu _1=\mu _{r_1 ,p,q,i}^{\xi _1\cup \xi _2} \end{aligned}$$

is the random-cluster measure on $r_1$ with boundary conditions $\xi _1\cup \xi _2$ and

$$\begin{aligned} \mu _2=\left( \mu _{r_2,p,q,i}^{\xi _2} \left\| \right. A\left( \xi _1 \right) \right) \Big |_{r_1} \end{aligned}$$

is the restriction to $r_1$ of the random-cluster with boundary conditions $\xi _2$ conditioned on the event $A\left( \xi _1 \right) ,$

$$\begin{aligned} \mu _1 {\mathop {=}\limits ^{d}}\mu _2 \,. \end{aligned}$$

Likewise, setting

$$\begin{aligned} \overline{\mu }_1=\mu _{r_1 ,p,q,i}^{\overline{\xi _1\cup \xi _2}} \end{aligned}$$

and

$$\begin{aligned} \overline{\mu }_2=\left( \mu _{r_2,p,q,i}^{\overline{\xi _2}} \left\| \right. A\left( \xi _1 \right) \right) \Big |_{r_1}\,, \end{aligned}$$

we have

$$\begin{aligned} \overline{\mu }_1 {\mathop {=}\limits ^{d}} \overline{\mu }_2 \,. \end{aligned}$$

### Proof

We first prove the equality $\mu _1 {\mathop {=}\limits ^{d}} \mu _2.$ As a preliminary step, we reduce to the case where the second set of boundary conditions are free. By Proposition 9 we can replace $\xi _2$ with boundary conditions $\xi _2'$ that do not contain any plaquettes outside of a larger box $r_3.$ Set $\mu _3=\mu _{r_3,p,q,i}^{\textbf{f}}.$ Assuming the result for free boundary conditions, we have that

$$\begin{aligned} \mu _1 {\mathop {=}\limits ^{d}}\left( \mu _3 \Big |_{r_1} \left\| \right. A\left( \xi _1 \cup \xi _2' \right) \right) \end{aligned}$$

and

$$\begin{aligned} \mu _2{\mathop {=}\limits ^{d}}\left( \left( \mu _3 \left\| \right. A\left( \xi _2' \right) \right) \Big |_{r_2} \left\| \right. A\left( \xi _1 \right) \right) \Big |_{r_1}\,, \end{aligned}$$

which coincide.

Now, assume that $\xi _2=\emptyset .$ Let $P_1$ be a subcomplex of $r_1,$ let $P_2$ (respectively, $P_3$) be the percolation subcomplexes of $r_2$ (respectively, $\mathbb {Z}^d$) containing all open plaquettes of $P_1$ and $\xi _1.$ $P_2$ and $P_3$ thus have the same *i*-plaquettes, but different $(i-1)$-skeleta. For $1\le j < k \le 3$ let $\phi _{k,j}^*:H^{i-1}\left( P_k;\;\mathbb {Z}_q \right) \rightarrow H^{i-1}\left( P_j;\;\mathbb {Z}_q \right)$ be the map on cohomology induced by the inclusion $\phi ^{k,j}:P_j\hookrightarrow P_k.$ $\phi _{3,2}^*$ is surjective and $\phi _{3,1}^*=\phi _{3,2}^* \circ \phi _{2,1}^*$ so

$$\begin{aligned} \left| {{\,\textrm{im}\,}}\phi _{3,1}^*\right| =\left| {{\,\textrm{im}\,}}\phi _{2,1}^*\right| \,. \end{aligned}$$

It follows that that

$$\begin{aligned} \mu _1\left( P_1 \right) \propto p^{\left| P_1\right| }\left( 1-p \right) ^{\left| P_1^c\right| } \left| {{\,\textrm{im}\,}}\phi ^{1,3}_*\right| =p^{\left| P_1\right| }\left( 1-p \right) ^{\left| P_1^c\right| } \left| {{\,\textrm{im}\,}}\phi _{2,1}^*\right| \end{aligned}$$

and

$$\begin{aligned} \mu _2\left( P_1 \right) =\mu _{r_2,p,q,i}^{\textbf{f}}\left( P_2 \right)&\propto p^{\left| P_2\right| }\left( 1-p \right) ^{\left| P_2^c\right| }\left| {{\,\textrm{im}\,}}\phi _{3,2}^*\right| \\&\propto p^{\left| P_1\right| }\left( 1-p \right) ^{\left| P_1^c\right| } \left| H^{i-1}\left( P_2;\;\mathbb {Z}_q \right) \right| \, \end{aligned}$$

where we have removed a factor that does not depend on $P_1.$

It suffices to show that

3 $$\begin{aligned} \left| H^{i-1}\left( P_2;\;\mathbb {Z}_q \right) \right| / \left| {{\,\textrm{im}\,}}\phi ^{2,1}_*\right| \end{aligned}$$

does not depend on the state of $P_1.$ Towards that end, we apply the long exact sequence of the pair $(P_2,P_1)$ (see page 199 of [8]; more detail is given for the homological analogue on page 115): 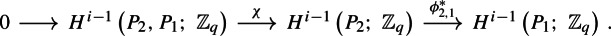 The leftmost term — corresponding to to $H^{i-2}\left( P_1;\;\mathbb {Z}_q \right)$ — vanishes because $P_1$ is a percolation subcomplexes. By exactness, $\chi$ is injective and

$$\begin{aligned} \ker \left( \phi _{2,1}^* \right) ={{\,\textrm{im}\,}}\chi \cong H^{i-1}\left( P_2,P_1;\;\mathbb {Z}_q \right) \,. \end{aligned}$$

We claim that $H^{i-1}\left( P_2,P_1;\;\mathbb {Z}_q \right)$ does not depend on the states of *i*-plaquettes of $P_1.$ Recall that $C^j\left( P_2,P_1;\;\mathbb {Z}_q \right)$ is is the group of *j*-cochains of $P_2$ that vanish on chains supported on $P_1,$ the relative coboundary map

$$\begin{aligned} \delta ^{j}_{P_2,P_1}: C^{j}\left( P_2,P_1;\;\mathbb {Z}_q \right) \rightarrow C^{j+1}\left( P_2,P_1;\;\mathbb {Z}_q \right) \end{aligned}$$

is the restriction of the usual coboundary map, and

$$\begin{aligned} H^{i-1}\left( P_2,P_1;\;\mathbb {Z}_q \right) =\ker \delta ^{i-1}_{P_2,P_1}/{{\,\textrm{im}\,}}\delta ^{i-2}_{P_2,P_1}\,. \end{aligned}$$

The $(i-1)$-skeletons of $P_2$ and $P_1$ do not depend on the state of their *i*-plaquettes, so neither do $C^{i-1}\left( P_2,P_1 \right)$ nor $C^{i-2}\left( P_2,P_1 \right) .$ It follows that ${{\,\textrm{im}\,}}\delta ^{i-2}_{P_2,P_1}$ and $\ker \delta ^{i-1}_{P_2,P_1}$ are also independent of $P_1$ (changing the codomain of a map does not change its kernel), and thus $H^{i-1}\left( P_2,P_1;\;\mathbb {Z}_q \right)$ is as well, completing the proof of the first statement.

We use Proposition 9 as a shortcut to prove that $\overline{\mu }_1 {\mathop {=}\limits ^{d}} \overline{\mu }_2.$ Choose a box $r_3\supset r_2$ large enough so that

$$\begin{aligned} \mu _{r_2 ,p,q,i}^{\overline{\xi }_2} {\mathop {=}\limits ^{d}} \mu _{r_2 ,p,q,i}^{\hat{\xi _2}_{r_3}} \end{aligned}$$

and

$$\begin{aligned} \mu _{r_1 ,p,q,i}^{\overline{\xi }_1} {\mathop {=}\limits ^{d}} \mu _{r_1 ,p,q,i}^{\hat{\xi _1\cup \xi _2}_{r_3}}. \end{aligned}$$

Since $\hat{\xi _1\cup \xi _2}_{r_3}=\xi _1\cup \hat{\xi _2},$ the desired statement follows from the one from non-wired boundary conditions. $\square$

## Duality and Boundary Conditions

We now consider boundary conditions in dual complexes. Recall that complex that we have defined on $\mathbb {Z}^d$ has an associated dual complex $\left( \mathbb {Z}^d \right) ^{\bullet } :=\mathbb {Z}^d + \left( 1/2,1/2,\ldots ,1/2 \right) .$ Each *i*-cell of $\mathbb {Z}^d$ intersects exactly one $(d-i)$-cell of $\left( \mathbb {Z}^d \right) ^{\bullet },$ so an *i*-dimensional percolation complex comes with a complementary dual $(d-i)$-dimensional dual complex. Let *r* be a box, let *P* be an *i*-dimensional percolation subcomplex of *r*,  and let *Q* be the dual complex. Recall our convention that *r* does not contain any boundary *i*-plaquettes and includes the entire $(i-1)$-skeleton. Also, *Q* is a subcomplex of $\overline{r^{\bullet }},$ by which we mean that it is allowed to contain $(d-i)$-plaquettes in the boundary. For convenience, set $\overline{Q}=Q\cup \partial r^{\bullet }.$ We will explore the relationship between $H^{i-1}\left( P;\;\mathbb {Z}_q \right)$ and $H^{d-i-1}\left( \overline{Q};\;\mathbb {Z}_q \right)$ by expressing them both in terms of $H_{i-1}\left( P;\;\mathbb {Z} \right) .$

We begin by recalling a few standard topological tools. Homology and cohomology groups with different coefficient groups are related by what is called the *universal coefficient theorem*, which has a version for homology and cohomology (Theorems 3A.3 and 3.2 in [8] respectively).

We first consider the case of homology and cohomology with coefficients in $\mathbb {Z}.$ $H_j\left( P;\;\mathbb {Z} \right)$ is a finitely generated abelian group so we may write

$$\begin{aligned} H_j\left( P;\;\mathbb {Z} \right) \cong \mathbb {Z}^{\textbf{b}_j\left( X \right) }\oplus T_j\left( X \right) \end{aligned}$$

where the Betti number $\textbf{b}_j\left( X \right)$ is the rank of $H_j\left( P;\mathbb {Z} \right)$ and the torsion subgroup $T_j\left( X \right)$ is finite. The universal coefficient theorem for cohomology yields that

4 $$\begin{aligned} H^j\left( X;\;\mathbb {Z} \right) \cong \left( H_j\left( X;\;\mathbb {Z} \right) /T_j \right) \oplus T_{j-1}\left( X \right) \, \end{aligned}$$

(this is stated as Corollary 3.3 in [8]).

If *P* is a percolation subcomplex of a box *r* in $\mathbb {Z}^d$ then the only homology and cohomology groups that depend on the state of *P* are in degree $\left( i-1 \right)$ and *i*. Since *P* does not contain any $\left( i+1 \right)$-cells, $H_i\left( P;\;\mathbb {Z} \right) \cong Z_i\left( P;\;\mathbb {Z} \right) =\ker \partial$ so $T_i\left( P \right) =0.$

To see what happens with $\mathbb {Z}_q$ coefficients, we apply the following equation, which is a consequence of the universal coefficient theorem for homology:

5 $$\begin{aligned} H_j\left( P;\mathbb {Z}_q \right) \cong \left( H_j\left( P;\;\mathbb {Z} \right) \otimes \mathbb {Z}_q \right) \oplus \textrm{Tor}\left( H_{j-1}\left( P;\;\mathbb {Z} \right) ,\mathbb {Z}_q \right) \end{aligned}$$

where $\textrm{Tor}\left( H,\mathbb {Z}_q \right)$ is the kernel of the map $g_q:H\rightarrow H$ given by $h\mapsto qh.$

For example, if $\mathbb {R}P^2$ is the real projective plane then $H_2\left( \mathbb {R}P^2;\;\mathbb {Z} \right) \cong \mathbb {Z}_2$ so $H_2\left( \mathbb {R}P^2;\;\mathbb {Z}_q \right) \cong \mathbb {Z}_q/\left( 2\mathbb {Z}_q \right)$ which is $\mathbb {Z}_2$ when *q* is even and zero when *q* is odd. This matches the computation performed in Sect. 2.

We also use a formulation of Alexander duality for percolation complexes previously given in [2].

### Proposition 11

Fix $0<i<d$ and a box *r* in $\mathbb {Z}^d.$ If *P* is a percolation subcomplex of $\overline{r}$ (*r*), *Q* is the dual complex, and $r'$ is the box $r^{\bullet }$ (respectively $\overline{r^{\bullet }}$) then there is an isomorphism

$$\begin{aligned} \mathcal {I} : H_{i}\left( P_r;\;\mathbb {Z} \right) \rightarrow H^{d-i-1}\left( Q\cup \partial r';\; \mathbb {Z} \right) \end{aligned}$$

where $H_j\left( X;\;\mathbb {Z} \right)$ and $H^j\left( X;\;\mathbb {Z} \right)$ denote the *j*-dimensional reduced homology and the *j*-dimensional reduced cohomology of *X* with integral coefficients.

Combining these facts yields the following proposition.

### Proposition 12

Let $$q\in \mathbb {N}+1.$$ Then

$$\begin{aligned} H^{i-1}\left( P;\;\mathbb {Z}_q \right) \cong \mathbb {Z}_q^{\textbf{b}_{i-1}\left( P;\;\mathbb {Z} \right) } \oplus \textrm{Tor}\left( H_{i-1}\left( P;\;\mathbb {Z} \right) , \mathbb {Z}_q \right) \,. \end{aligned}$$

and

$$\begin{aligned} \tilde{H}^{d-i-1}\left( \overline{Q};\;\mathbb {Z}_q \right) \cong \mathbb {Z}_q^{\textbf{b}_{i}\left( P;\;\mathbb {Z} \right) } \oplus \textrm{Tor}\left( H_{i-1}\left( P;\;\mathbb {Z} \right) , \mathbb {Z}_q \right) \,. \end{aligned}$$

In particular, there is a constant $c=c\left( N,i,d \right)$ so that

6 $$\begin{aligned} \left| H^{i}\left( P;\;\mathbb {Z}_q \right) \right| =\left| H^{d-i-1}\left( \overline{Q};\;\mathbb {Z}_q \right) \right| q^{c-\left| P\right| }\,. \end{aligned}$$

### Proof

For the first claim,

$$\begin{aligned} H^{i-1}\left( P;\;\mathbb {Z}_q \right)&\cong H_{i-1}\left( P;\;\mathbb {Z}_q \right)  &   \text {Proposition } 2\\&\cong H_{i-1}\left( P;\;\mathbb {Z} \right) \otimes \mathbb {Z}_q  &   (5), H_{i-2}\left( P;\;\mathbb {Z} \right) \cong 0 \\&\cong \mathbb {Z}_q^{\textbf{b}_{i-1}\left( P;\;\mathbb {Z} \right) } \oplus \textrm{Tor}\left( H_{i-1}\left( P;\;\mathbb {Z} \right) , \mathbb {Z}_q \right)  &   \text {properties of } \otimes \,. \end{aligned}$$

We now demonstrate the second claim.

$$\begin{aligned} H^{d-i-1}\left( \overline{Q};\;\mathbb {Z}_q \right)&\cong H_{d-i-1}\left( \overline{Q};\;\mathbb {Z}_q \right)  &   \text {Proposition } 2 \\&\cong \left( H_{d-i-1}\left( \overline{Q};\;\mathbb {Z} \right) \otimes \mathbb {Z}_q \right)  &   (5), H_{d-i-2}\left( \overline{Q};\;\mathbb {Z} \right) \cong 0 \\&\cong \left( H^{i}\left( P;\;\mathbb {Z} \right) \otimes \mathbb {Z}_q \right)  &   \text {Corollary } 11 \\&\cong \mathbb {Z}_q^{\textbf{b}_{i}\left( P;\;\mathbb {Z} \right) } \oplus \textrm{Tor}\left( H_{i-1}\left( P;\;\mathbb {Z} \right) , \mathbb {Z}_q \right)  &   (4), \text { properties of } \otimes \,. \end{aligned}$$

Finally, $H_0\left( P;\;\mathbb {Z} \right) \cong \mathbb {Z},$ $H_i\left( P;\;\mathbb {Z} \right) ,$ and $H_{i-1}\left( P;\;\mathbb {Z} \right)$ are the only non-zero homology groups of *P*,  so the Euler–Poincaré theorem (Theorem 2.44 in [8]) yields that

$$\begin{aligned} \chi \left( P \right) =1+(-1)^{i-1} \textbf{b}_{i-1}\left( P;\;\mathbb {Z} \right) +(-1)^i\textbf{b}_{i}\left( P;\;\mathbb {Z} \right) =\left| P\right| +\sum _{j=0}^{i-1}\left| P^{(j)}\right| \,. \end{aligned}$$

Then (6) follows because the number of *j*-dimensional plaquettes in *P* ($\left| P^{(j)}\right|$) does not depend on *P* for $j\le i-1.$ $\square$

The following special case of Alexander duality is reproduced from [2].

### Proposition 13

Fix $0<i<d$ and a box *r* in $\mathbb {Z}^d.$ If *P* is a percolation subcomplex of $\overline{r}$ (*r*), *Q* is the dual complex, and $r'$ is the box $r^{\bullet }$ (respectively $\overline{r^{\bullet }}$) then there is an isomorphism

$$\begin{aligned} \mathcal {I} : H_{i}\left( P_r;\;\mathbb {Z} \right) \rightarrow H^{d-i-1}\left( Q\cup \partial r';\; \mathbb {Z} \right) \end{aligned}$$

where $H_j\left( X;\;\mathbb {Z} \right)$ and $H^j\left( X;\;\mathbb {Z} \right)$ denote the *j*-dimensional reduced homology and the *j*-dimensional reduced cohomology of *X* with integral coefficients.

We are now ready to prove that the *i*-dimensional PRCM with free boundary conditions on *r* is dual to a $(d-i)$-dimensional wired PRCM on $\overline{r^{\bullet }}.$ The argument is similar to the one used in [10], with the key difference being the use of the preceding proposition.

### Theorem 14

Let $q\in \mathbb {N}+1$ and $1\le i \le d-1.$ Also, define

7 $$\begin{aligned} p^*=p^*(p,q)= \frac{\left( 1-p \right) q}{\left( 1-p \right) q + p}\,. \end{aligned}$$

Then, if *r* is a box in $\mathbb {Z}^d,$

$$\begin{aligned} \mu ^{\textbf{f}}_{r,p,q,i}\left( P \right) = \mu ^{\textbf{w}}_{\overline{r^\bullet },p^*,q,d-i}\left( Q \right) \ \end{aligned}$$

and

$$\begin{aligned} \mu ^{\textbf{f}}_{\overline{r},p,q,i}\left( P \right) = \mu ^{\textbf{w}}_{r^\bullet ,p^*,q,d-i}\left( Q \right) \ \end{aligned}$$

### Proof

In order to show the first claim, we compute

$$\begin{aligned} \mu ^{\textbf{f}}_{r,p,q,i}\left( P \right)&=\frac{1}{\tilde{Z}}p^{\left| P\right| }\left( 1-p \right) ^{\left| r^{(i)}\right| -\left| P\right| }\left| H^{i-1}\left( P;\;\mathbb {Z}_q \right) \right| \\&= \frac{\left( 1-p \right) ^{\left| r^{(i)}\right| }}{\tilde{Z}}\left( \frac{p}{1-p} \right) ^{\left| P\right| }\left| H^{i-1}\left( P;\;\mathbb {Z}_q \right) \right| \\&= \frac{\left( 1-p \right) ^{\left| r^{(i)}\right| }}{\tilde{Z}}\left( \frac{p}{1-p} \right) ^{\left| P\right| }\left| H^{d-i-1}\left( \overline{Q};\;\mathbb {Z}_q \right) \right| q^{c-\left| P\right| }  &   (6)\\&= \frac{q^{c}\left( 1-p \right) ^{\left| r^{(i)}\right| }}{\tilde{Z}}\left( \frac{q(1-p)}{p} \right) ^{-\left| P\right| }\left| H^{d-i-1}\left( \overline{Q};\;\mathbb {Z}_q \right) \right| \\&= \frac{q^{c}\left( 1-p \right) ^{\left| r^{(i)}\right| }}{\tilde{Z}}\left( \frac{q(1-p)}{p} \right) ^{\left| \overline{Q}\right| -\left| r^{(i)}\right| }\left| H^{d-i-1}\left( \overline{Q};\;\mathbb {Z}_q \right) \right| \\&= \frac{q^{c}\left( 1-p \right) ^{\left| r^{(i)}\right| }}{\tilde{Z}}\left( \frac{p^*}{1-p^*} \right) ^{\left| \overline{Q}\right| -\left| r^{(i)}\right| }\left| H^{d-i-1}\left( \overline{Q};\;\mathbb {Z}_q \right) \right|  &   (7)\\&= \frac{q^{c}\left( 1-p \right) ^{\left| r^{(i)}\right| }}{\left( p^* \right) ^{\left| r^{(i)}\right| }\tilde{Z}}\frac{\left( p^* \right) ^{\left| \overline{Q}\right| }}{\left( 1-p^* \right) ^{\left| \overline{Q}\right| - \left| r^{(d-i)}\right| }}\left| H^{d-i-1}\left( \overline{Q};\;\mathbb {Z}_q \right) \right| \\&:=\frac{1}{\tilde{Z}^\bullet }\left( p^* \right) ^{\left| \overline{Q}\right| }\left( 1-p^* \right) ^{\left| r^{(d-i)}\right| - \left| Q\right| }\left| H^{d-i-1}\left( \overline{Q};\;\mathbb {Z}_q \right) \right| \\&= \mu ^{\textbf{w}}_{r^\bullet ,p^*,q,d-i}\left( Q \right) \,. \end{aligned}$$

The proof of the second claim is nearly identical, but uses the parenthetical formulation of Proposition 13. $\square$

Since our notion of boundary conditions corresponds to a fixed external percolation complex, a natural set of dual boundary conditions is given by the dual complex. More precisely, for a box *r* in $\mathbb {Z}^d$ and boundary conditions $\xi ,$ the dual measure on $\overline{r^{\bullet }}$ is defined by setting $\xi ^{\bullet }$ to include all $(d-i)$-plaquettes dual to closed *i*-plaquettes of $\xi .$ All of the pieces are now in place to describe the distribution of the dual complex to the PRCM with boundary conditions.

### Theorem 15

$$\begin{aligned} \mu ^{\xi }_{r,p,q,i}\left( P \right) = \mu ^{\overline{\xi ^\bullet }}_{\overline{r^{\bullet }},p^*,G,d-i}\left( Q \right) \end{aligned}$$

### Proof

By Proposition 9, we can choose a box $r_2$ large enough so that

$$\begin{aligned} \mu _{r ,p,q,i}^{\xi } {\mathop {=}\limits ^{d}} \left( \mu _{r_2,p,q,i}^{\textbf{f}} \left\| \right. A\left( \xi \right) \right) \Big |_{r} \end{aligned}$$

and

$$\begin{aligned} \mu _{\overline{r^{\bullet }} ,p^*,G,d-i}^{\overline{\xi ^{\bullet }}} {\mathop {=}\limits ^{d}} \left( \mu _{\overline{r_2^{\bullet }},p^*,G,d-i}^{\textbf{w}} \left\| \right. A\left( \xi ^{\bullet } \right) \right) \Big |_{\overline{r^{\bullet }}}\,. \end{aligned}$$

We can conclude by applying Theorem 14. $\square$

As a corollary, we have that $\left| {{\,\textrm{im}\,}}\phi ^*\right| =\left| {{\,\textrm{im}\,}}\psi _*^{\textrm{BM}}\right| .$ This identity can be proven directly using a commutative diagram involving long exact sequences of the pairs $(P_\xi ,P)$ and $(\overline{Q}\cup \overline{Q_{\xi ^{\bullet }}},\overline{Q})$ together with Poincaré and Lefschetz dualities. Then one could prove Proposition 9 using the same argument as in Theorem 14. However, the resulting proof would be longer.

## The Infinite Volume Limit

In this section, we apply our previous results to understand the random complexes on $\mathbb {Z}^d$ constructed as weak limits of finite volume PRCMs with boundary conditions. First, we recall some basic tools to compare different measures. For two measures $\mu _1$ and $\mu _2$ on percolation subcomplexes, we say that $\mu _1$ is *stochastically dominated* by $\mu _2$ and write $\mu _1 \le _{\textrm{st}}\mu _2$ if there is a coupling $\kappa$ of random complexes $P_1$ and $P_2$ distributed according to $\mu _1$ and $\mu _2$ respectively so that

$$\begin{aligned} \kappa \left( P_1 \subset P_2 \right) = 1. \end{aligned}$$

The main tool we will use to show stochastic domination is due to Holley [11].

### Theorem 16

(Holley’s Inequality) Let *I* be a finite index set and let

$$\begin{aligned} \left( X_i \right) _{i \in I},\left( Y_i \right) _{i \in I} \in \left\{ 0,1\right\} ^I \end{aligned}$$

be random vectors distributed according to strictly positive probability measures $\mu _1$ and $\mu _2.$ Suppose that for each pair $\left( W_i \right) _{i \in I},\left( Z_i \right) _{i \in I} \in \left\{ 0,1\right\} ^I$ with $W_i \le Z_i$ for each $j \in I,$

$$\begin{aligned}&\mu _1\left( X_j = 1 : X_i = W_i \text { for all } i \in I \setminus \left\{ j\right\} \right) \\&\qquad \le \mu _2\left( Y_j = 1 : Y_i = Z_i \text { for all } i \in I \setminus \left\{ j\right\} \right) \,. \end{aligned}$$

Then $$\mu _1 \le _{\textrm{st}}\mu _2.$$

We can now compare measures in subcomplexes in the free and wired cases.

### Lemma 17

Let $X \subset Y$ be subcomplexes of $\mathbb {Z}^d.$ Then $\mu ^{\textbf{f}}_{X,p,q,i} \le _{\textrm{st}}\left( \mu ^{\textbf{f}}_{Y,p,q,i} \right) \big |_{X}$ and $\left( \mu ^{\textbf{w}}_{Y,p,q,i} \right) \big |_X \le _{\textrm{st}}\mu ^{\textbf{w}}_{X,p,q,i}.$

### Proof

For any *i*-plaquette $\sigma \in X^{\left( i \right) }$ and any percolation subcomplex $P \subset Y,$ if

$$\left| H^{i-1}\left( P \cup \sigma ;\;\mathbb {Z}_q \right) \right| < \left| H^{i-1}\left( P \cap X;\;\mathbb {Z}_q \right) \right| $$

then

$$\left| H^{i-1}\left( \left( P \cap X \right) \cup \sigma ;\;\mathbb {Z}_q \right) \right| < \left| H^{i-1}\left( P \cap X;\;\mathbb {Z}_q \right) \right| \,.$$

On the other hand, if

$$\left| H^{i-1}\left( P \cup \partial Y \cup \sigma ;\;\mathbb {Z}_q \right) \right| = \left| H^{i-1}\left( P \cup \partial Y \cap X;\;\mathbb {Z}_q \right) \right| $$

then

$$\left| H^{i-1}\left( \left( P \cap X \right) \cup \partial X \cup \sigma ;\;\mathbb {Z}_q \right) \right| = \left| H^{i-1}\left( \left( P \cap X \right) \cup \partial X;\;\mathbb {Z}_q \right) \right| \,.$$

The desired stochastic domination is then a consequence of Theorem 16. $\square$

This gives a quick proof that the free and wired limits exist.

### Proposition 18

The limits

$$\begin{aligned} \mu _{\mathbb {Z}^d,p,q,i}^{\textbf{f}} :=\lim _{n \rightarrow \infty } \mu ^{\textbf{f}}_{\Lambda _n,p,q,i}\, \end{aligned}$$

and

$$\begin{aligned} \mu _{\mathbb {Z}^d,p,q,i}^{\textbf{w}} :=\lim _{n \rightarrow \infty } \mu ^{\textbf{w}}_{\Lambda _n,p,q,i}\, \end{aligned}$$

exist.

### Proof

By Lemma 17, for fixed *m*,  $\left( \mu ^{\textbf{f}}_{\Lambda _n,p,q,i} \right) \big |_{\Lambda _m}$ and $\left( \mu ^{\textbf{w}}_{\Lambda _n,p,q,i} \right) \big |_{\Lambda _m}$ are increasing and decreasing in *n* respectively, so they must converge. $\square$

In the classical RCM, it is a standard fact that the free and wired boundary conditions are extremal, since they are the boundary conditions that maximize and minimize the number of connected components respectively. The analogous statement for the PRCM is true for similar reasons.

### Proposition 19

Let *r* be a box in $\mathbb {Z}^d$ and let $\xi$ be any boundary conditions. Then

$$\begin{aligned} \mu ^{\textbf{f}}_{r,p,q,i} \le _{\textrm{st}}\mu ^{\xi }_{r,p,q,i} \le _{\textrm{st}}\mu ^{\textbf{w}}_{r,p,q,i}\,. \end{aligned}$$

In addition, for any $\mu _{\mathbb {Z}^d,p,q,i}$ which is an infinite volume random-cluster measure which is a weak limit of measures on boxes with boundary conditions,

8 $$\begin{aligned} \mu ^{\textbf{f}}_{\mathbb {Z}^d,p,q,i} \le _{\textrm{st}}\mu _{\mathbb {Z}^d,p,q,i} \le _{\textrm{st}}\mu ^{\textbf{w}}_{\mathbb {Z}^d,p,q,i}\,. \end{aligned}$$

As a consequence of Theorem 15, there is also a relationship between dual limiting measures, when they exist.

### Theorem 20

Suppose the weak limit

$$\begin{aligned} \mu ^\xi _{\mathbb {Z}^d,p,q,i} :=\lim _{n \rightarrow \infty } \mu ^{\xi }_{\Lambda _n,p,q,i} \end{aligned}$$

exists. Then the weak limit

$$\begin{aligned} \mu ^{\overline{\xi ^{\bullet }}}_{\left( \mathbb {Z}^d \right) ^{\bullet },p^*,q,d-i} :=\lim _{n \rightarrow \infty } \mu ^{\overline{\xi ^{\bullet }}}_{\overline{\Lambda _n^{\bullet }},p^*,q,d-i} \end{aligned}$$

also exists, and satisfies

$$\begin{aligned} \mu ^\xi _{\mathbb {Z}^d,p,q,i}\left( P \right) {\mathop {=}\limits ^{d}} \mu ^{\overline{\xi ^{\bullet }}}_{\left( \mathbb {Z}^d \right) ^{\bullet },p^*,q,d-i}\left( Q \right) \,. \end{aligned}$$

We also see that the general free and wired measures are dual.

### Corollary 21

The free and wired measures $\mu ^{\textbf{f}}_{\mathbb {Z}^d,p,q,i}$ and $\mu ^{\textbf{w}}_{\mathbb {Z}^d,p,q,i}$ satisfy

$$\begin{aligned} \mu ^{\textbf{f}}_{\mathbb {Z}^d,p,q,i} \left( P \right) {\mathop {=}\limits ^{d}} \mu ^{\textbf{w}}_{\mathbb {Z}^d,p^*,q,d-i}\left( Q \right) \end{aligned}$$

### Proof

The statement essentially follows from Theorem 20. Since the choice between Definitions 5 and 7 is irrelevant for wired boundary conditions, we need only check that

$$\begin{aligned} \lim _{n\rightarrow \infty } \mu ^{\textbf{w}}_{\overline{\Lambda _n},p,q,i} {\mathop {=}\limits ^{d}} \lim _{n\rightarrow \infty } \mu ^{\textbf{w}}_{\Lambda _n,p,q,i}\,. \end{aligned}$$

This follows from Lemma 17, since for all *n*,  we have

$$\begin{aligned} \Lambda _n \subset \overline{\Lambda _n} \subset \Lambda _{n+1}\,. \end{aligned}$$

$$\square $$

## Uniqueness of the Infinite Volume Measure

In the classical RCM with a fixed parameter *q*,  the wired and free infinite volume measures are known to coincide except possibly at a countable set of values of *p*. In this section we adapt Grimmett’s proof of this result [5] to the PRCM; only minor modifications are required. One application of this result comes from Proposition 34 in [2]: if *p* is such that there is an infinite volume PRCM, two notions of surface tension given in terms of the asymptotic probability that an $(i-1)$-cycle $\gamma$ is null-homologous coincide. When $q\in \mathbb {N}+ 2,$ this in turn implies that two definitions of Wilson loop tension agree in the coupled PLGT.

Fix $1 \le i \le d,$ let *r* be a box, let $\xi$ be boundary conditions and let $\Omega _r$ be the set of *i*-dimensional percolation subcomplexes on *r*. We work with a slight modification of the partition function for the PRCM on *r* with boundary conditions $\xi ,$ namely

$$\begin{aligned} Y^{\xi }_{r,p,q}&:=\left( 1-p \right) ^{-\left| r^{\left( i \right) }\right| }Z^{\xi }_{r,p,q} = \left( 1-p \right) ^{-\left| r^{\left( i \right) }\right| }\sum _{P \in \Omega _r} p^{\left| P\right| }\left( 1-p \right) ^{\left| r^{\left( i \right) }\right| -\left| P\right| }\left| {{\,\textrm{im}\,}}\phi ^*\right| \\&= \sum _{P \in \Omega _r} \left| {{\,\textrm{im}\,}}\phi ^*\right| \exp \left( \pi \left| P\right| \right) \,, \end{aligned}$$

where $Z^{\xi }_{r,p,q}$ is the usual partition function and $\pi :=\log \left( p/\left( 1-p \right) \right) .$ We now consider a notion of pressure. For a box *r* and boundary conditions $\xi$, write

$$\begin{aligned} f^{\xi }_r\left( p,q \right) :=\frac{1}{\left| r^{\left( i \right) }\right| } \log Y^{\xi }_{r,p,q}\,. \end{aligned}$$

### Proposition 22

Let $\left\{ r_k\right\}$ be an increasing sequence of boxes with $\bigcup _k r_k = \mathbb {Z}^d$ and let $\xi$ be a set of boundary conditions. Then the limit

$$\begin{aligned} f\left( p,q \right) = f^{\xi }\left( p,q \right) :=\lim _{k \rightarrow \infty } f^{\xi }_{r_k}\left( p,q \right) \end{aligned}$$

exists and does not depend on the choice of $\left\{ r_k\right\}$ or $\xi .$ Moreover, $f\left( p,q \right)$ is a convex function of $\pi$ and therefore differentiable as a function of $p \in \left( 0,1 \right)$ except possibly on a countable set.

### Proof

We first verify that the limit $f^{\textbf{f}}\left( p,q \right)$ exists and does not depend on $\left\{ r_k\right\}$. For simplicity of presentation, we will show this in the case of cubes and leave the extension to general boxes as an exercise. Note that if $\Lambda _m=\left[ -m,m \right] ^d,$ we have for each $0 \le j \le d$ that

$$\begin{aligned} \lim _{m \rightarrow \infty } \frac{\left| \Lambda _m^{\left( j \right) }\right| }{m^d}=2^d\left( {\begin{array}{c}d\\ j\end{array}}\right) \end{aligned}$$

so $\lim _{m \rightarrow \infty } f^{\textbf{f}}_{\Lambda _m}\left( p,q \right)$ exists if and only if

$$\begin{aligned} \lim _{m \rightarrow \infty } \frac{1}{m^d} Y^{\textbf{f}}_{\Lambda _m,p,q} = -\log \left( 1-p \right) \lim _{m \rightarrow \infty } \frac{1}{m^d} Z^{\textbf{f}}_{\Lambda _m,p,q} \end{aligned}$$

does.

We now recall a standard tool for understanding the topology of a space from that of its subspaces. The *Mayer–Vietoris Sequence for cohomology* relates the cohomology of a union of two spaces to the cohomology groups of the spaces and their intersection. Denote $P\cap Y$ by $P_Y.$ The Mayer–Vietoris sequence in full generality can be found in Sections 2.2 and 3.2 of [8], but here we will just describe what it yields for a decomposition of the form $P_X = P_A \cup P_B$ induced by a decomposition $X = A \cup B.$ Then the following sequence is exact, meaning that the image of one map in the sequence is the kernel of the next: 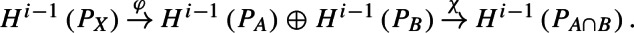


Now by the first isomorphism theorem,

$$\begin{aligned} \frac{\left| H^{i-1}\left( P_A;\;\mathbb {Z}_q \right) \right| \left| H^{i-1}\left( P_B;\;\mathbb {Z}_q \right) \right| }{\left| H^{i-1}\left( P_X;\;\mathbb {Z}_q \right) \right| }\le \left| H^{i-1}\left( P_{A\cap B};\;\mathbb {Z}_q \right) \right| \end{aligned}$$

with equality holding if *A* and *B* are disjoint. We also have for any subcomplex $Y \subset X,$

$$\begin{aligned} \left| H^{i-1}\left( Y;\;\mathbb {Z}_q \right) \right| \le q^{\left| Y^{\left( i-1 \right) }\right| }\,. \end{aligned}$$

Now, assume that the *i*-skeleta of *A* and *B* are disjoint. Combining the previous inequalities and summing over the *i*-plaquettes of $A\cup B$ yields

9 $$\begin{aligned} \frac{1}{q^{\left| A\cap B^{\left( i-1 \right) }\right| }} Z^{\textbf{f}}_{A,p,q}Z^{\textbf{f}}_{B,p,q} \le Z^{\textbf{f}}_{A \cup B,p,q} \end{aligned}$$

Now let $\Lambda _n \subset \Lambda _m.$ Consider a maximal packing of $\Lambda _m$ by disjoint translates $\left\{ \Lambda ^l\right\} _{l=1}^{k}$ of $\Lambda _n$ where

$$\begin{aligned} \left( \frac{m}{n+1}-1 \right) ^d\le k=k\left( m,n \right) \end{aligned}$$

Denote the union of these translates by *A* and note that the disjointness of the cubes implies that

10 $$\begin{aligned} Z^{\textbf{f}}_{A,p,q}=\left( Z^{\textbf{f}}_{\Lambda _n,p,q} \right) ^k\,. \end{aligned}$$

Let *B* be the induced subcomplex of $$\Lambda _n$$ containing all *i*-plaquettes that are not in any of the $$\Lambda ^l.$$ *B* is contained in the union of $$m^d-n^d k $$ unit *d*-cubes so for any subcomplex $$B_0$$ of *B* and any $$1\le j\le d$$

$$\begin{aligned} \left| B_0^{\left( j \right) }\right| \le c_j\left( m^d-n^d k \right) \,, \end{aligned}$$

where $$c_j :=2^{2d-j}\left( {\begin{array}{c}d\\ j\end{array}}\right) .$$ As a consequence,

$$\begin{aligned} \log \left( Z^{\textbf{f}}_{B,p,q} \right) \le \left| B^{\left( i-1 \right) }\right| \le c_{i-1}\log \left( q \right) \left( m^d-n^d k \right) \,. \end{aligned}$$

Then, by Equations 9 and 10, for fixed *n*

$$\begin{aligned}&\frac{1}{m^d}\log \left( Z^{\textbf{f}}_{\Lambda _m,p,q} \right) \\&\qquad \ge \frac{1}{m^d}\left( k \log \left( Z^{\textbf{f}}_{\Lambda _n,p,q} \right) +\log \left( Z^{\textbf{f}}_{B,p,q} \right) - \left| A\cap B^{\left( i-1 \right) }\right| \log \left( q \right) \right) \\&\qquad \ge \left( \frac{1}{n+1}-\frac{1}{m} \right) ^d\log \left( Z^{\textbf{f}}_{\Lambda _n,p,q} \right) -c_{i-1}\log \left( q \right) \left( 1-\left( \frac{n}{n+1}-\frac{n}{m} \right) ^d \right) \,. \end{aligned}$$

So

$$\begin{aligned}&\liminf _{m\rightarrow \infty } \frac{1}{m^d}\log \left( Z^{\textbf{f}}_{\Lambda _m,p,q} \right) \\&\qquad \begin{aligned} \ge \limsup _{n\rightarrow \infty } \liminf _{m\rightarrow \infty }&\left[ \left( \frac{1}{n+1}-\frac{1}{m} \right) ^d \log \left( Z^{\textbf{f}}_{\Lambda _n,p,q} \right) \right. \\&\quad \left. -c_{i-1}\log \left( q \right) \left( 1-\left( \frac{n}{n+1}-\frac{n}{m} \right) ^d \right) \right] \end{aligned}\\&\qquad \begin{aligned}\ge \limsup _{n\rightarrow \infty } \left[ \frac{1}{\left( n+1 \right) ^d} \log \left( Z^{\textbf{f}}_{\Lambda _n,p,q} \right) -c_{i-1}\log \left( q \right) \left( 1-\left( \frac{n}{n+1} \right) ^d \right) \right] \end{aligned}\\&\begin{aligned}\qquad =\limsup _{n\rightarrow \infty } \frac{1}{n^d}\log \left( Z^{\textbf{f}}_{\Lambda _n,p,q} \right) \end{aligned}\,, \end{aligned}$$

and $$f^{\textbf{f}}\left( p,q \right) $$ exists.

Since $\left| H^{i-1}\left( \partial r_k;\;\mathbb {Z}_q \right) \right| = q^{\left| \left( \partial r_k \right) ^{\left( i \right) }\right| -1},$ we have

$$\begin{aligned} Y^{\textbf{f}}_{r_k,p,q} \le Y^{\xi }_{r_k,p,q} \le Y^{\textbf{w}}_{r_k,p,q} \le Y^{\textbf{f}}_{r_k,p,q}q^{\left| \left( \partial r_k \right) ^{\left( i \right) }\right| -1}\,. \end{aligned}$$

Notice that

$$\begin{aligned} \frac{\log \left( q^{\left| \left( \partial r_k \right) ^{\left( i \right) }\right| } \right) }{\left| r_k^{\left( i \right) }\right| } \xrightarrow []{k \rightarrow \infty } 0 \end{aligned}$$

so the existence of the limit $f^{\textbf{f}}\left( p,q \right)$ implies that $f\left( p,q \right)$ exists and is well defined. As is done for the classical RCM in the proof of Theorem 4.58 of [12], taking the second derivative of $f^{\textbf{f}}_{r_k}$ shows that it is a convex function of $\pi ,$ so the limiting function $f\left( p,q \right)$ is also convex. Since $\pi$ is a differentiable function of *p*,  we therefore have that $f\left( p,q \right)$ is also differentiable outside of a countable set. $\square$

### Theorem 23

Suppose $f\left( p,q \right)$ is differentiable as a function of *p* at $p = p_0.$ Then there is a unique infinite volume PRCM with parameters $p_0,q.$

### Proof

Let $\xi \in \left\{ \textbf{f},\textbf{w}\right\}$ and let $h^{\xi }\left( p,q \right) = \mu ^{\xi }_{\mathbb {Z}^d,p,q,i}\left( \sigma \text { is open} \right) .$ Since both measures are easily checked to be translation invariant, this value does not depend on the choice of plaquette $\sigma .$ We now compare $h^{\textbf{f}}\left( p,q \right)$ and $h^{\textbf{w}}\left( p,q \right) .$ Notice that for any box *r*, 

11 $$\begin{aligned} \frac{d f^{\xi }_r\left( p,q \right) }{d \pi } = \frac{1}{\left| r^{\left( i \right) }\right| } \mathbb {E}_{\mu ^{\xi }}\left( \left| \left\{ \sigma \in r^{\left( i \right) } \text{ open }\right\} \right| \right) \,. \end{aligned}$$

Then for any $\sigma _0 \in r^{\left( i \right) },$ it follows from translation invariance and Proposition 8 that

$$\begin{aligned} \frac{1}{\left| r^{\left( i \right) }\right| } \mathbb {E}_{\mu ^{\textbf{f}}}\left( \left| \left\{ \sigma \in r^{\left( i \right) } \text{ open }\right\} \right| \right)&\le \mu ^{\textbf{f}}_{\mathbb {Z}^d,p,q,i}\left( \sigma _0 \text { is open} \right) \\&\le \mu ^{\textbf{w}}_{\mathbb {Z}^d,p,q,i}\left( \sigma _0 \text { is open} \right) \\&\le \frac{1}{\left| r^{\left( i \right) }\right| } \mathbb {E}_{\mu ^{\textbf{w}}}\left( \left| \left\{ \sigma \in r^{\left( i \right) } \text{ open }\right\} \right| \right) \,. \end{aligned}$$

Write $\pi _0 = \log \left( p_0/\left( 1-p_0 \right) \right) .$ Now from Proposition 22 and convexity,

$$\begin{aligned} \lim _{k \rightarrow \infty } \frac{d f^{\xi }_{r_k}\left( p,q \right) }{d \pi } \left( \pi _0 \right) = \frac{d f\left( p,q \right) }{d \pi } \left( \pi _0 \right) \,, \end{aligned}$$

so it follows that $h^{\textbf{f}}\left( p_0,q \right) = h^{\textbf{w}}\left( p_0,q \right) .$ Since $\mu ^{\textbf{f}}_{\mathbb {Z}^d,p_0,q,i} \le _{\textrm{st}}\mu ^{\textbf{w}}_{\mathbb {Z}^d,p_0,q,i}$ by Proposition 19, a standard comparison of cylinder events (Proposition 4.6 in [12]) gives

$$\begin{aligned} \mu ^{\textbf{f}}_{\mathbb {Z}^d,p_0,q,i} \,{\buildrel d \over =}\, \mu ^{\textbf{w}}_{\mathbb {Z}^d,p_0,q,i}\,, \end{aligned}$$

and all measures at $p_0$ coincide. $\square$

## Positive Association in the PRCM

In this section we give an alternative proof using algebraic topology of the result of [3] that the PRCM is positively associated. First we adapt the proof of [4] for the PRCM with coefficients in a field.

### Theorem 24

Let *X* be a finite cubical complex. Then $\mu _{X,p,q,i}$ is positively associated in the sense that for any two increasing events *A* and *B*

$$\begin{aligned} \mu _{X,p,q,i}\left( A\cap B \right) \ge \mu _{X,p,q,i}\left( A \right) \mu _{X,p,q,i}\left( B \right) . \end{aligned}$$

### Proof

By Theorem 16 it suffices to show that

12 $$\begin{aligned} \mu _{X,p,q,i}\left( P\cup P' \right) \mu _{X,p,q,i}\left( P\cap P' \right) \ge \mu _{X,p,q,i}\left( P \right) \mu _{X,p,q,i}\left( P' \right) \end{aligned}$$

for any $P,P'.$ As

$$\begin{aligned} \left| P\cup P'\right| +\left| P\cap P'\right| =\left| P\right| +\left| P'\right| \end{aligned}$$

the desired statement will follow from

$$\begin{aligned} \left| H^{i-1}\left( P \cap P' \right) \right| \left| H^{i-1}\left( P \cup P' \right) \right| \ge \left| H^{i-1}\left( {P} \right) \right| \left| H^{i-1}\left( {P'} \right) \right| \,, \end{aligned}$$

where cohomology is taken with coefficients in $\mathbb {Z}_q.$

Consider the following part of the Mayer–Vietoris sequence for *P* and $P':$
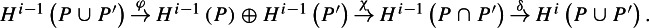


We apply the first isomorphism theorem for abelian groups (that is, that if $\phi :G\rightarrow H$ is a homomorphism then $G\cong {{\,\textrm{im}\,}}\phi \oplus \ker \phi$) to the first three terms of the sequence.

First,

$$\begin{aligned} H^{i-1}\left( P\cup P' \right) \cong {{\,\textrm{im}\,}}\varphi \oplus \ker \varphi \end{aligned}$$

so

$$\begin{aligned} \left| H^{i-1}\left( P\cup P' \right) \right| =\left| {{\,\textrm{im}\,}}\varphi \right| \left| \ker \varphi \right| . \end{aligned}$$

Next,

$$\begin{aligned} H^{i-1}\left( P \right) \oplus H^{i-1}\left( P' \right) \cong {{\,\textrm{im}\,}}\chi \oplus \ker \chi \cong {{\,\textrm{im}\,}}\chi \oplus {{\,\textrm{im}\,}}\varphi \end{aligned}$$

and

$$\begin{aligned} \left| H^{i-1}\left( P \right) \right| \left| H^{i-1}\left( P' \right) \right| =\left| H^{i-1}\left( P \right) \oplus H^{i-1}\left( P' \right) \right| =\left| {{\,\textrm{im}\,}}\chi \right| \left| {{\,\textrm{im}\,}}\varphi \right| \,. \end{aligned}$$

A similar computation yields

$$\begin{aligned} \left| H^{i-1}\left( P\cap P' \right) \right| =\left| {{\,\textrm{im}\,}}\chi \right| \left| {{\,\textrm{im}\,}}\delta \right| \,. \end{aligned}$$

Combining these results gives

$$\begin{aligned} \left| H^{i-1}\left( P \cap P' \right) \right| \left| H^{i-1}\left( P \cup P' \right) \right|&= \left| {{\,\textrm{im}\,}}\chi \right| \left| {{\,\textrm{im}\,}}\delta \right| \left| {{\,\textrm{im}\,}}\varphi \right| \left| \ker \varphi \right| \\&\ge \left| {{\,\textrm{im}\,}}\chi \right| \left| {{\,\textrm{im}\,}}\varphi \right| \\&= \left| H^{i-1}\left( {P} \right) \right| \left| H^{i-1}\left( {P'} \right) \right| \,, \end{aligned}$$

as desired. $$\square $$

### Corollary 25

The measures $\mu ^{\xi }_{r,p,q,i}$ and $\mu ^{\overline{\xi }}_{r,p,q,i}$ are also positively associated, as are the limiting infinite volume PRCMs, if they exist.

### Proof

Let $\mu _r$ be a measure of the form $\mu ^{\xi }_{r,p,q,i}$ or $\mu ^{\overline{\xi }}_{r,p,q,i}.$ By Proposition 9 and Corollary 10 for a sufficiently large cube $\Lambda \supset r$ we have that

$$\begin{aligned} \mu _r{\mathop {=}\limits ^{d}}\left( \mu _{\Lambda } \Vert A \right) \Big |_{r} \end{aligned}$$

where *A* is the event that the states of all plaquettes in $\Lambda \setminus r$ agrees with those of $\xi .$ It is easy to see that Equation 12 for $\mu _\Lambda$ implies it for $\mu _r.$

The positive association for infinite volume measures follows from that of the finite volume measures by Proposition 4.10 of [12]. $\square$

## Data Availability

This manuscript has no associated data.
